# Prevalence of HTLV-1/2 and HIV-1/2 in individuals attending Public Health Center in Mozambique. One HTLV-1 symptomatic case report

**DOI:** 10.1186/1742-4690-8-S1-A69

**Published:** 2011-06-06

**Authors:** Adele Caterino-de-Araujo, Mariana C Magri, Emanuela A S Costa, Rolanda C R Manuel

**Affiliations:** 1 Centro de Imunologia, Instituto Adolfo Lutz, Coordenadoria de Controle de Doenças da Secretaria de Estado da Saúde de São Paulo, Brazil; 2 Faculdade de Ciências Farmacêuticas – USP, São Paulo, SP, Brazil; 3 Hospital Central de Maputo, Universidade Eduardo Mondlane, Maputo, Mozambique

## Background

In the last decades, an outbreak of HIV/AIDS occurred in Mozambique (MZ), and since HIV and HTLV share the same routes of virus transmission/acquisition, we decided to estimate the actual prevalence of such retroviruses.

## Materials and methods

Blood samples were collected from 208, 226 and 318 individuals from Northern, Central and Southern MZ, respectively, of all socioeconomic and demographic strata, attending Public Health Centers. Sera were assessed for HIV-1/2- and HTLV-1/2–specific antibodies by enzyme immunoassays and confirmed by Western blot. HTLV-1/2-seropositive blood samples were submitted for PCR (LTR, env, and pol) and real-time PCR (pol) assays, and sequenced.

## Results

An overall HTLV-1/2 prevalence of 2.3% was observed, and increased with age. Regional variation in the prevalence of HIV and HTLV-1/2 was observed; 32.2%, 65.5% and 44% of individuals tested HIV-positive in Northern, Central and Southern MZ, respectively, and 2.4%, 3.9% and 0.9% tested HTLV-1-positive in the same regions. No association between HTLV-1 infection and socio-demographic variables or HIV status was detected. One woman aged 54 years, born in Northern MZ, HIV-negative, who complaints of rheumatic pain, presented high HTLV-1 proviral load, and phylogenetic analysis of env and LTR sequences clustered HTLV-1-isolate into Cosmopolitan subtype, transcontinental subgroup A (GenBank Accession numbers HM770441 and JF271853).

## Discussion and conclusions

These data suggest different risk factors and epidemiologic correlates of HIV and HTLV-1 transmission in MZ. HTLV-1 is endemic in this country, and arthritis was associated with HTLV-1 infection in one patient.

